# From Global to Local and Vice Versa: On the Importance of the ‘Globalization’ Agenda in Continental Groundwater Research and Policy-Making

**DOI:** 10.1007/s00267-016-0722-2

**Published:** 2016-06-18

**Authors:** Viachaslau Filimonau, Johannes A. C. Barth

**Affiliations:** 1 Faculty of Management, Bournemouth University, Talbot Campus, Fern Barrow, Poole, Dorset BH12 5BB UK; 2 Department of Geography and Geosciences, Friedrich-Alexander University Erlangen-Nuremberg (FAU), GeoZentrum Nordbayern, Schlossgarten 5, 91054 Erlangen, Germany

**Keywords:** Groundwater, Freshwater, Sustainability, Water resources management, Assessment

## Abstract

Groundwater is one of the most important environmental resources and its use continuously rises globally for industrial, agricultural, and drinking water supply purposes. Because of its importance, more knowledge about the volume of usable groundwater is necessary to satisfy the global demand. Due to the challenges in quantifying the volume of available global groundwater, studies which aim to assess its magnitude are limited in number. They are further restricted in scope and depth of analysis as, in most cases, they do not explain how the estimates of global groundwater resources have been obtained, what methods have been used to generate the figures and what levels of uncertainty exist. This article reviews the estimates of global groundwater resources. It finds that the level of uncertainty attached to existing numbers often exceeds 100 % and strives to establish the reasons for discrepancy. The outcome of this study outlines the need for a new agenda in water research with a more pronounced focus on groundwater. This new research agenda should aim at enhancing the quality and quantity of data provision on local and regional groundwater stocks and flows. This knowledge enhancement can serve as a basis to improve policy-making on groundwater resources globally. Research-informed policies will facilitate more effective groundwater management practices to ensure a more rapid progress of the global water sector towards the goal of sustainability.

## Introduction

Groundwater is an increasingly important environmental resource as a large share of global economy and 2.1 billion of people rely on it as the primary source of freshwater (United Nations Children’s Fund-UNICEF and World Health Organisation 2012). Nonetheless, the importance of groundwater is often underestimated, while its role in the global and regional water balances is poorly quantified (Gleeson et al. 2015; Llamas 2003; Margat and van der Gun 2013). This is particularly alarming because limited understanding of the groundwater value is attributed not only to the general public, but also to environmental managers and policy-makers, water industry professionals, and academics who work in the field of water resources and environmental management (Lavoie et al. 2013; Margat and van der Gun 2013; Pandey et al. 2011; van der Gun 2012). One reason for this lack of knowledge is that groundwater occurs as an invisible environmental resource (Gleeson et al. 2012). The ‘out of sight, out of mind’ principle may have determined the yet inadequate attention paid to the issue of groundwater on a global scale.

While groundwater is one of the most widespread environmental resources, its availability does not often match the demand (Gleeson et al. 2012). Given the global significance of groundwater, it is essential to pay more attention to the issues of its occurrence, distribution, stocks, and flows (Margat and van der Gun 2013; Wada et al. 2014). It is equally important to educate the general public, industry representatives, policy-makers, and academia about the fragile and complex nature of local, regional, and global groundwater resources. This would enable better protection and facilitate more sustainable consumption and management practices.

The situation is gradually changing and effective groundwater management represents a growing research stream. Recently, particular attention has been paid to the issues of groundwater abstraction, overexploitation, and pollution (Holman and Trawick 2011; Sparks et al. 2015; Wada et al. 2010). There is a general consensus in the literature that reliable estimates of groundwater resources are crucial to tackle these issues (Healy 2010). This is because effective mitigation can only be developed if accurate figures on groundwater stocks and flows become available (Fazal et al. 2001). Furthermore, groundwater represents a cross-boundary resource that can affect significant territories (Margat and van der Gun 2013). Therefore, groundwater quantity and quality assessments should be approached from both, local/regional and global, scales. Despite the challenges attributed to producing a global outlook on groundwater resources, it helps identify major opportunities for management. Global assessments can also be scaled down to regional and local levels (Cash and Moser 2000). This approach follows a key principle of sustainable development ‘think globally, act locally,’ which suggests that global knowledge is necessary in order to design local mitigation actions (Agenda 21 1992).

A literature review indicates that accurate and reliable figures of global groundwater resources are difficult to obtain. First, there is limited research on global groundwater estimates in general and many studies have focused on surface water, aiming to quantify and assess its global availability (Gleeson et al. 2012, 2015). Second, groundwater research in industrialized countries has more been concerned with the issue of groundwater quality, rather than quantity (Balderacchi et al. 2013). This approach needs rethinking because the problems of groundwater quality and its availability are interlinked and management of the increasing global demand for freshwater will rely on a combination of both (Mays 2013).

Further analysis shows that the available literature often fails to explain how existing estimates of global groundwater resources have been obtained (Margat and van der Gun 2013). In many cases, this is manifested by the fact that figures retrieved from previous studies are taken as a ‘given,’ without questioning their accuracy and reliability of the original source. Better understanding of how the estimates of global groundwater resources have been produced is crucial as this enables better and more critical assessment of their reliability and facilitates evaluation of the factors that might contribute to uncertainty.

Another important issue is attributed to significant differences between existing figures of global groundwater resources (Margat and van der Gun 2013). This calls for more research on this topic, to help identify the most accurate estimate and outline the pathways to reduce the magnitude of uncertainty. Better understanding of existing estimates of global groundwater resources is vital because accurate values are necessary to foster more sustainable groundwater management practices. They should enhance the effectiveness of water management-related policies globally.

This study aims to demonstrate the global importance of groundwater as an environmental resource by highlighting its role in the global water balance (“Global Water Resources: Defining Stocks and Flows” section). It also strives to discuss its economic and societal value (“Economic and Societal Significance of Global Groundwater Resources” section). The study further reviews existing estimates of global groundwater resources as reported in specialist literature, examines how these have been produced, establishes the range of uncertainty attributed to them, and evaluates the implications for sustainable groundwater management and policy-making (“Global (Ground) Water Estimates: A Critical Review” section). It discusses measures that should be applied to enhance the accuracy of global groundwater estimates and puts forward recommendations for their implementation (“Towards More Effective Management of Groundwater Resources: The Importance of ‘Globalization’ of Groundwater Research and Policy-Making Agenda” section).

### Groundwater as a Pivotal Environmental Resource

#### Global Water Resources: Defining Stocks and Flows

**Fig. 1:**
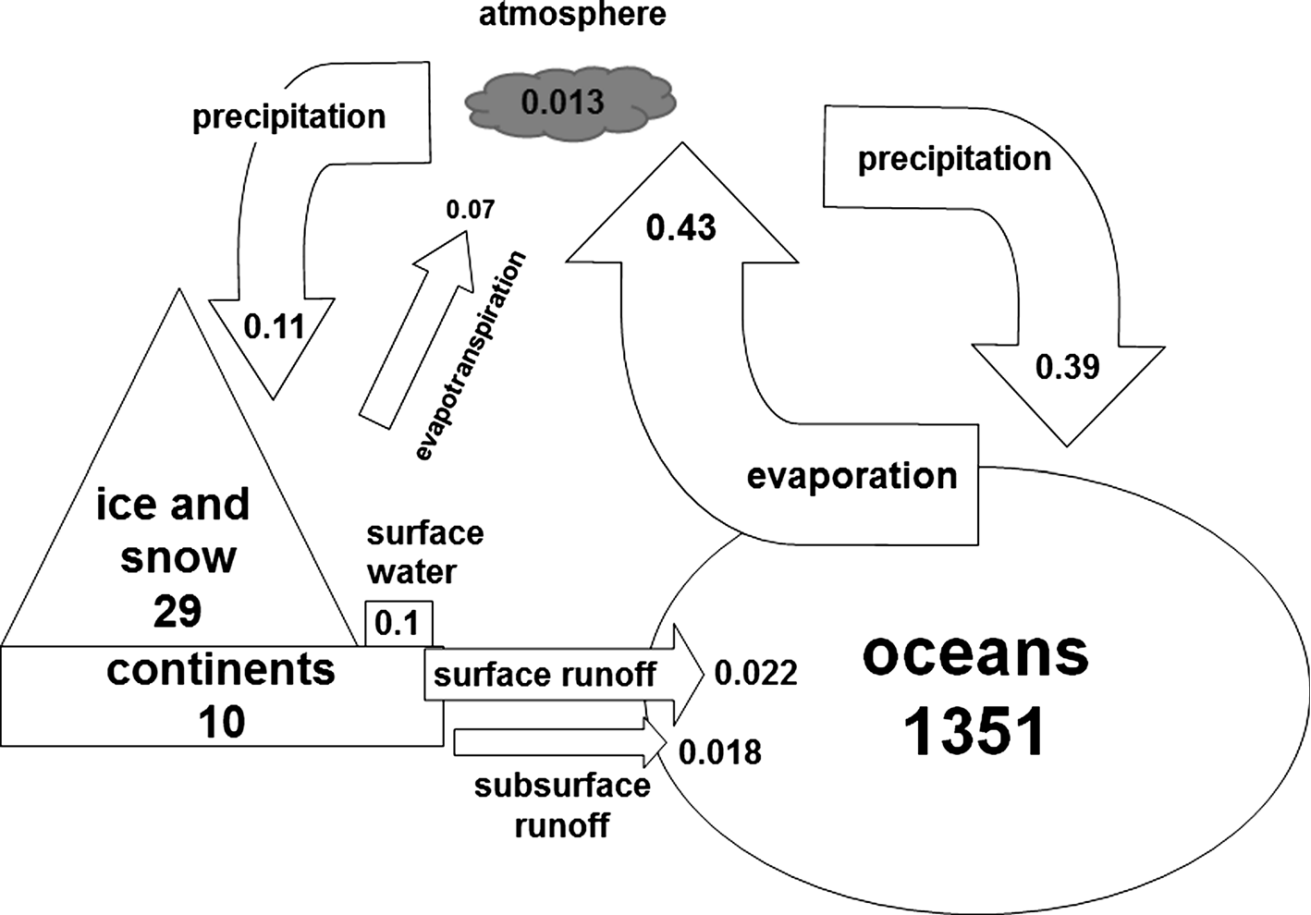
Global water balance, schematic, and not-to-scale. Water stocks are in million km^3^ and water flows are in million km^3^ a^−1^. The key sources for this figure are Berner and Berner (1996), Seiler and Gat (2007), UNEP (2008), and World Business Council for Sustainable Development—WBCSD (2005)

According to Margat and van der Gun (2013), stock is one of the two variables that characterize global water resources. The second variable is flow or the rate of water exchange between water storage compartments. The combination of both is often termed the annual global water balance (Berner and Berner 1996). This paper presents average numbers from a literature review on the global water balance (Fig. 1). While this review strives to be comprehensive, it cannot be considered complete. It is limited to the number of studies that were identified via a search on the citation-based academic search platform, Google Scholar, conducted within the period of 4–7th April 2016. The following search terms were used: *global water balance* and *global groundwater stocks OR flows OR resources*. The OR operator enabled inclusion of literature sources that dealt with global groundwater resources in general and global groundwater stocks and flows in particular. In order to limit this analysis, only the first 1000 search results revealed by Google Scholar were analysed. No filter was applied to the search outcome to benefit from the transparency and comprehensiveness of the search algorithm employed by this platform (Harzing and van der Wal 2008).

The total global stock of surface and close-to-surface water that is not involved in mantle processes is estimated between 1300 and 1500 million km^3^ (Fig. 1). While this figure is significant, the usability of global water resources is limited. This is primarily because saline ocean water represents about 1250–1465 million km^3^ or 96–97.5 % of the total global water volume. While saline water is increasingly seen as a resource for future water supply (Post et al. 2013), its usability is yet constrained by economic and technological factors (Greenlee et al. 2009). The remaining 2.5–4 % or 35–50 million km^3^ of global water are more crucial as this volume is attributed to freshwater resources the usability of which is not restricted by salinity. Global freshwater is subdivided into ice and snow (~2–3 % of globally available water), surface water as well as groundwater (~0.5–1 %). The volumes of water stored in the atmosphere account for 0.013 km^3^, thus making up only 0.00093 % of global water resources (Berner and Berner 1996; Shiklomanov 1996; Shiklomanov and Rodda 2003; United Nations Environment Programme-UNEP 2008; United Nations Educational, Scientific and Cultural Organisation-UNESCO 2003)^1^

In terms of flows, annual global precipitation represents the largest water flow on Earth with the magnitude of approximately 0.5 million km^3^ a^−1^. Almost 80 % (0.39 million km^3^ a^−1^) of this volume is attributed to precipitation over the ocean. Evapotranspiration from land surface is averaged with 0.07 million km^3^ a^−1^. Assuming a steady-state system, the difference between continental precipitation and continental evapotranspiration (0.11–0.07 million km^3^ a^−1^) represents the global runoff from the continents (0.04 million km^3^ a^−1^). The global runoff can be subdivided into the surface runoff (estimated as 55 % of the total runoff or 0.022 million km^3^ a^−1^) and the subsurface runoff that mostly consists of groundwater discharge into the ocean (estimated as 45 % or 0.018 million km^3^ a^−1^) (Seiler and Gat 2007). The sum of the groundwater and surface water discharge to the ocean (0.04 million km^3^ a^−1^) also makes up the difference between evaporation from the oceans (0.43 million km^3^ a^−1^) and precipitation over the oceans (0.39 million km^3^ a^−1^). The latter closes the cycle of the annual global water balance.

In terms of usability, the volume of global freshwater is of primary interest. While ice and snow represent the key storage compartment for freshwater on Earth and their melt water is a major feed source for global rivers, the direct use of ice and snow is constrained by a number of factors, such as geographical availability. In contrast, groundwater has a more ubiquitous geographical distribution and usually requires little treatment before its use. This underlines the strategic importance of groundwater as a global freshwater stock.

#### Economic and Societal Significance of Global Groundwater Resources

Globally, groundwater represents a vital environmental resource, both in terms of stocks and flows. From the utility point of view, the role of groundwater is equally important. With the annual abstraction rate of about 0.001 million km^3^ in 2010, it accounts for about 25 % of the annual global freshwater withdrawal (UNESCO 2012). Worldwide, this makes groundwater the most used environmental resource, ahead of gravel, coal, and crude oil (Zektser and Everett 2004).

Global freshwater withdrawal is predicted to grow in order to meet the rising demands from the industry, agriculture and households (Alcamo et al. 2003; Wada et al. 2010). For instance, the numbers by UNESCO (2012) suggest that, in 2011, the volume of freshwater withdrawn globally had reached 0.004 million km^3^. For comparison, this amount was equivalent to approximately 17 % of the volume of Lake Baikal, the world’s largest open freshwater reservoir, with a volume of about 0.024 million km^3^. In 2025, annual global freshwater withdrawals are predicted to reach about 0.005 million km^3^ (Seiler and Gat 2007; Shiklomanov and Rodda 2003; WBCSD 2005). This number corresponds to about 21 % of the volume of Lake Baikal. By 2050, the global demand for freshwater is expected to grow up to 0.012 million km^3^ (Nature 2008) which is equivalent to the volume of Lake Superior or about 50 % of the volume of Lake Baikal. Importantly, the relative contribution of groundwater abstraction to global freshwater withdrawals may also grow due to the increasingly high dependence of the world population and global economy on groundwater resources (Wada et al. 2010, 2014).

The current figures of global fresh- and groundwater withdrawals indicate that they do not exceed the magnitude of the annual global freshwater (0.04 million km^3^ a^−1^) and groundwater (0.018 million km^3^ a^−1^) flow. This also holds true for future projections up to 2050 (UNESCO 2012; Wada et al. 2010). These figures do not however indicate the differences in annual groundwater withdrawals across the world. They should therefore be taken with caution as they may lead to the incorrect conclusion that there is enough useable groundwater everywhere. This is not true because the volumes of the groundwater withdrawn have significant geographical variations and in many arid regions the magnitude of groundwater withdrawals exceeds the rates of the annual groundwater renewal (Goldenberg 2014). This often implies unsustainable groundwater management that may lead to long-term detrimental environmental and socio-economic effects.

In terms of economic and societal role, Foster and Chilton (2003) stated that groundwater globally provides about 50 % of drinking water, 40 % of industrial water, and 20 % of the water used for irrigation. In a more recent study, Margat and van der Gun (2013) provide different figures which suggest that 70 % of all groundwater abstracted in the world is intended for irrigation, 21 % for domestic use (which includes drinking water), and 9 % for industrial purposes. This discrepancy may be partially attributed to the variations in methodological approaches that were applied to calculate the share of groundwater. It is more likely, however, that it arises due to the lack of monitoring of groundwater extraction across the world, especially in developing countries, which hampers derivation of accurate estimates globally. This underlines the issue of insufficient attention paid to groundwater in research and policy-making, both at a local/regional and a global scale.

According to UNICEF and the World Health Organisation (2012), today about 30 % of the world’s population relies on groundwater with expected rapid growth in the future. The above emphasises the necessity to pay closer attention to groundwater stocks and flows. In a first instance, this should aim to produce more accurate numbers of local and regional stocks and their associated flows. A second step should aim to comprehend the magnitude of local and regional extractions with subsequent reinforcements of sustainable extraction levels.

In summary, although annual global freshwater and groundwater withdrawals do not exceed the rates of the annual freshwater flow and large reservoirs of freshwater exist, projected shortages of available good-quality freshwater are expected to intensify in the future (see, for example, UNEP 2008). This is because freshwater replenishment, flows, and stocks are unevenly distributed on Earth. While arid regions suffer most, even the areas with sufficient water renewal rates may increasingly become affected by water shortages if the hygienic and chemical quality of water deteriorates. In addition, the global demand for freshwater is likely to increase in the future due to the population growth, agricultural, and industrial development and the overall rise of living standards among the population. Among available water resources, groundwater currently represents the most plausible water resource for consumption as it is fairly easy to access, relatively inexpensive to mine and often requires little or no further treatment. Better understanding of how much groundwater can be withdrawn without imposing a detrimental effect on the environment is essential. A fundamental prerequisite for this aim is the accurate figures on globally available groundwater stocks and flows.

### Global (Ground)Water Estimates: A Critical Review

Analysis of literature shows that the number of original estimates of global (ground)water stocks and flows is limited by the figures reported in a handful of studies (Table 1). Most of the sources reviewed do not clarify how the estimates have been produced. Furthermore, many of the figures cited are taken for ‘granted’ from past research.

**Table 1:**
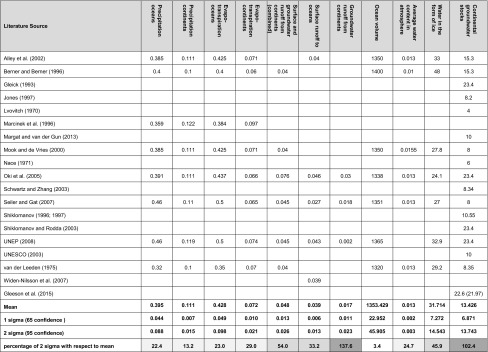
Estimates of flows and stocks within the different elements of the annual global water balance as reported in literature

Numbers are in million km^3^ a^−1^ for flows and in million km^3^ for stocks. Note that recent work by Gleeson et al. (2015) also differentiates groundwater ages

Further analysis reveals significant uncertainties across the estimates (Table 1). It suggests that, when a normal distribution is assumed, among all elements of the global water balance, the level of uncertainty lies within a 10 % range only for the estimates of water stored in the oceans (Table 1). This is largely because the volume of surface water-containing reservoirs should be fairly easy to calculate given the ongoing advancements in geophysical and echosound methods (see, for example, Charette and Smith 2010).

In contrast, the water flows partaking in the global water balance in the form of precipitation and evapotranspiration alongside the volume of the water stored in the atmosphere are characterized by considerably higher levels of uncertainty of up to 30 % (Table 1, marked in *light grey*). This is primarily due to the fact that the accurate evaluations of precipitation and evaporation over the oceans are hardly possible, which results in higher uncertainties (Joyce et al. 2004). Uncertainties in assessing the magnitude of the water stored in the atmosphere may also be attributed to the difficulties in determining its volume because of short residence times (Berner and Berner 1996).

Further analysis shows that the estimates of water stored in the form of ice and snow as well as surface- and groundwater runoff from the continents (combined) have higher uncertainties (Table 1, marked in *medium-spectre grey*). Here, the surface runoff from the continents is only estimated for the largest rivers that have gauging stations. Smaller rivers, which in sum discharge considerable amounts of water to the oceans, are often not taken into account and this contributes to uncertainty (Berner and Berner 1996). This is because smaller rivers do not always host gauging stations (Verdin and Verdin 1999); furthermore, they may not necessarily provide sufficiently long and detailed discharge records, particularly in developing countries and remote regions, such as those adjacent to the Arctic Ocean (Peterson et al. 2002).

Most importantly, the estimates of global groundwater stocks and its runoff from the continents to the ocean are characterized by uncertainties of over 100 % (Table 1, marked in *dark grey*). It is further worth mentioning that only three literature sources (0.3 %) from the 1000 reviewed Google Scholar search results have provided figures on the groundwater runoff from the continents. This shows that global groundwater stocks and its runoff are the elements of the global water balance that remain difficult to quantify and their poor visibility and limited access make significant impacts on the accuracy of their assessment. The high levels of uncertainty attributed to these elements of the global water balance are of particular concern given the critical socio-economic role of increasing water use for irrigation, households, and the industry. Notably, some figures of global groundwater stocks vary considerably from the rest (i.e. 23.4 and 22.6 (21.97) million km^3^, Table 1). Closer analysis shows that this is because these larger numbers incorporate both saline- and fresh groundwater and do not differentiate between these two types of groundwater. This may lead to the incorrect conclusions about the magnitude of globally useable groundwater resources. It is argued that this figure should be revisited to display the amount of fresh groundwater only given that such references as UNEP publications are often used as key data sources by academics and non-academics, also from outside the field of groundwater resources management. However, even when the larger estimates of global groundwater stocks including saline- and freshwater are removed, the level of uncertainty attributed to this element of the global water balance remains at about 90 %. Significant uncertainty established for the estimates of global fresh groundwater stocks and flows can be attributed to the following factors.

First, some of the figures originate from the literature sources published from 1970 to 1990. This is also confirmed by the most recent analyses by Gleeson et al. (2015). The accuracy of groundwater assessment methods at the time may have been insufficient. This can partially explain the different numbers of global groundwater stocks associated with these sources. Nonetheless, authors such as Lvovitch (1970), Nace (1971), and Van der Leeden (1975) also serve as references in more recent studies on the global water balance (e.g. Linton 2004). In order to avoid incorrect conclusions regarding the magnitude of global groundwater stocks, future references should be given to more recent studies that are based on more advanced methods of assessment, such as modelling and remote sensing (see Sect. 3.1 *Methods to produce the estimates of global groundwater stocks and flows: an overview*).

Second, the high levels of discrepancy among more recent figures on global groundwater stocks and flows may result from the complexity of groundwater systems whose extent is difficult to measure. This includes hydrogeological characteristics of the surface and subsurface waters, climatic, and atmospheric processes and water regimes of surface water storage bodies (Balek 1989; Freeze and Cherry 1979; Polak et al. 2007; Seiler and Gat 2007). For example, large discrepancies exist in volumetric evaluations of the water stored in the subsurface when only visible piezometers and well data are used. This also holds true when high-resolution geophysical techniques are used because they often have limited spatial ranges of application, such as at the level of river basins (Günter et al. 2007a). Furthermore, data on groundwater recharge and subsurface runoff are sparse and can only be determined with hydrogeological gradients and control planes. This is especially difficult in developing countries that often have insufficient resources to deploy and maintain measurement networks of groundwater observation wells (Frimpong et al. 2003).

Third, the estimates of groundwater discharge to surface waters, particularly from coastal aquifers to the ocean, often rely on the numbers that represent the difference between the continental recharge via precipitation and the discharge via large rivers (Sophocleous 2002). Moreover, while groundwater is deemed to be present in the subsurface up to the depth of several kilometres, it can be considered as useable only to a few hundred metres (Berner and Berner 1996; Margat and van der Gun 2013). Beyond this depth, efforts for abstraction often render groundwater use uneconomic as the extraction costs alongside salinities increase. However, exact critical depths for groundwater use are difficult to determine. They often have significant geographical variations and show challenges in the assessment of pore space in aquifers. Likewise, while useable groundwater resources may exist beneath the ocean floor, little is known about their magnitude (Post et al. 2013; van Geldern et al. 2013).

Lastly, while the global water balance is predominantly controlled by natural factors such as evaporation, transpiration, precipitation, and runoff, recently human-induced impacts have also been influencing global groundwater stocks and flows. On larger scales, this occurs indirectly through the acceleration of climatic changes. Locally and regionally, the effect can be made through the reshaping of waterways (e.g. via damming, river channelling, and subsurface constructions), groundwater abstraction, irrigation, or by the reinjection of water into aquifers. To date, the impact of these factors on the global water balance has been difficult to quantify, especially in the long term. However, a number of examples demonstrate a detrimental effect of human activities on global (ground)water quantity, quality, and dynamics (Diamond 2005). These impacts are expected to intensify with population growth and therefore call for more attention. This is particularly important for groundwater whose capability to replenish is limited due to slow annual flows, long memory effects, and anticipated greater use.

#### Methods to Produce the Estimates of Global Groundwater Stocks and Flows: An Overview

Analysis of the estimates of global groundwater stocks and flows demonstrates that most literature sources do not report on the methods used for assessment of global groundwater resources. The lack of this information hampers critical evaluation of the reliability of the figures provided. Subsequent analysis of specialist literature was undertaken to establish how the estimates of global groundwater stocks and flows have been obtained with a view to reveal the key methods applied for assessment. The following methods have been identified: simplified volume calculations, modelling, remote sensing, geographic information systems (GIS), geophysical and geochemical (i.e. isotope) techniques, and surface water discharge determinations.

The primary method for estimating global groundwater stocks, which many of the subsequent studies have relied upon, was developed by Shiklomanov (1996, 1997). It generates a figure of groundwater stocks for each continent (except for Antarctica) by multiplying the total area of the continent by the estimated groundwater depth, a water loss factor, and the effective porosity (Jacobson 2000; Shiklomanov and Rodda 2003). A critical question for this method is to define the depth of groundwater storage that depends on a number of factors, such as hydrogeological structure, recharge hydrodynamics, rock types, and geothermal gradients. For example, porosity can only be roughly estimated, and not accurately measured, due to the small-scale complexity of the geological formations in the subsurface (Arnell 2002). Otherwise, it can also be determined in pumping tests the application of which is however mostly limited to the depth of several dozens of metres (Massmann and Madden 1994).

The maximum estimated depth of groundwater storage is taken by Shiklomanov (1996, 1997) as 2000 m. For this depth, the three zones of groundwater storage are distinguished on the basis of the differences in hydrodynamics. These zones are attributed different values of effective porosity that range from 5 %, for the lowest, to 15 %, for the uppermost, layer. Reflecting the differences in porosity, the uppermost zone of the subsurface across the globe stores 3.6 million km^3^, the intermediate depth zone accommodates 6.2 million km^3^ and the lowest zone hosts 13.6 million km^3^ of groundwater. When combined, this yields a total of 23.4 million km^3^. Shiklomanov (1996, 1997) further differentiates between saline and fresh groundwater stocks; the latter are estimated as 10.5 million km^3^; however, no explanation is provided on how this figure is obtained. While the estimates by Shiklomanov (1996, 1997) have been the basis for many subsequent studies on global groundwater stocks, it is important to recognize that significant changes have occurred since 1997 in terms of the methods developed to determine porosity values and define water loss factors. The latter may have been affected by climate change and human-induced impacts. This calls for the refinement of the method by Shiklomanov (1996, 1997) with a view to enhance its accuracy and generate more precise numbers of global groundwater stocks.

Newer estimates of global groundwater resources, particularly flows, are predominantly generated via models where the WaterGAP Global Hydrology Model (Döll et al. 2003), the Global Energy and Water Cycle Experiment Project (Potter and Colman 2003), and The Global Soil Wetness Project (Oki et al. 2005) represent the key analytical platforms. These approaches to groundwater assessment have their own uncertainties. For example, they assume that climate parameters are stationary. This means that the climatic patterns observed in the past will also hold true in the future with insignificant variations (Gleick 1993). This assumption cannot always be made with natural water dynamics and accelerated climatic changes. Groundwater assessments should ideally rely on continuous and dense data of geological, hydrological, and meteorological parameters which are essential for model calibration (Dragoni and Sukhija 2008; Shiklomanov and Rodda 2003). Such data can be difficult to compile on a global level, hence the local and regional focus becomes paramount. For instance, calibration of the WaterGAP model is based on the data that, currently, cover only 50 % of the global land area and 70 % of the actively discharging area (Döll and Fiedler 2008), which contributes to uncertainty. Most importantly, many large-scale hydrological models such as those by Arnell (1999), Klepper and van Drecht (1998), and Yates (1997) focus primarily on atmospheric and land surface waters and largely ignore groundwater. In these models, groundwater stocks and flows are usually treated as a residual element that therefore receives little attention.

Recently, remote sensing techniques have found increased application to evaluate the magnitude of spatial and temporal variations in water storage and flows on Earth. For example, the Gravity Recovery and Climate Experiment (GRACE) twin satellite project was initiated in 2002 (Rodell and Famiglietti 2002). It aims at monitoring changes in water storage in aquifers on a monthly basis and mapping their spatial distribution (Günter et al. 2007a,b; Jin and Feng 2014). The key feature of GRACE is its capability to establish the magnitudes of variations. Hence, it is best applied to assess the relative changes in water storage, such as the volume of the global groundwater recharge and discharge rates (Jin and Feng 2014). The capability of GRACE to provide absolute estimates of the water stored in different compartments of the global water balance is limited. Analysis of the literature indicates that it has not yet been utilized to specifically assess the magnitude of global groundwater stocks and flows. Furthermore, the project is still under development and its measurements are expensive (Günter et al. 2007b; Jin and Feng 2014). Lastly, the capability of GRACE to estimate short-term variations in water stocks and flows is limited. This may cause systematic errors due to tides, wave motion or fluctuations in global soil moisture (Günter et al. 2007a). This may diminish its value when applied on a local and a regional scale.

A number of studies rely on GIS application to groundwater-related issues, including assessments of groundwater resources. The primary focus of these studies is on local and regional scales (see, for example, Chen et al. 2005; Rahman 2008). Globally, the GIS method has been applied within the scope of the World-wide Hydrogeological Mapping and Assessment Programme (WHYMAP) and the International Groundwater Resources Assessment Center (IGRAC) (2015) (Struckmeier and Richts 2006). These projects are mainly concerned with providing better visualization of the outcome of smaller scale studies on the assessment of groundwater stocks. They do not however quantify or model the magnitude of global groundwater resources (IGRAC 2015; Struckmeier and Richts 2006, 2008). The GIS-based assessments of groundwater stocks and flows depend on reliable and high-frequency input data on surface and aquifer systems. Input of such localized and regionalized data often requires significant data mining and programming skills (Welsh 2007) which can be expensive and labour intense.

Recent work by Gleeson et al. (2015) used global distributions of siliciclastic, carbonate, volcanic, and crystalline rocks. They were plotted with their estimated porosity distribution versus depth to estimate groundwater volumes stored in the continental crust. They further used tritium data and two-dimensional models to outline groundwater ages of 25, 50, 75, and 100 years with the volumes of 0.19, 0.35, 0.45, and 0.63 million km^3^, respectively. This shows that the largest part of continental groundwater (i.e. 21.97 million km^3^) has ages of over 100 years and thus likely higher salinities.

In summary, several methods have been applied to produce estimates of global groundwater resources. The key limitation of these methods is that none of them have been developed to specifically address the groundwater issues and their focus has been on surface water stocks and flows. This calls for a change, given the increasingly significant role played by groundwater in global society and economy. It is argued that new and combined efforts need to be developed to raise the position of groundwater on political, societal, and research agendas. Existing estimates of global groundwater stocks and flows are characterized by significant levels of uncertainty and there is a clear need for more accurate assessments to ensure the development of more sustainable groundwater consumption and management practices. These assessments are best to be carried out at local and regional scales with subsequent scaling up to inform the international groundwater research and policy-making agenda.

### Towards More Effective Management of Groundwater Resources: The Importance of ‘Globalization’ of Groundwater Research and Policy-Making Agenda

The above analysis indicates that there is a need for a new and more groundwater-focused research agenda that should aim at enhancing data reliability on global groundwater resources. This can in part be achieved via the joint application of the most accurate and up-to-date methods of assessment. The approach adapted in some studies, which prioritizes surface waters, while treating groundwater as a residual element of analysis needs revision. Likewise, the approaches that concentrate on the evaluation of groundwater quality rather than quantity should be revisited.

The concept of ‘globalization’ should be closely integrated into groundwater research and policy-making. Here, the most reliable estimates of groundwater stocks and flows can be derived at local and regional scales. The extensive databases of local and regional data on groundwater should be developed and then utilized to inform research and policy-making agenda internationally. To this end, there is a need for close intra-country and intra-regional research collaboration on the topic of assessment of groundwater resources as aquifers rarely do align with national borders. Given that this relates to a number of political and social issues, the new research agenda should be interdisciplinary in nature and aim to unite experts from the political, socioeconomic, and environmental dimensions.

#### Global Groundwater Data: Access and Retrieval

There is a need for more empirical studies that enhance the quality of data on global groundwater. Margat and van der Gun (2013) claim that these data are difficult to access across the world which hampers any comparative analysis of the volumes of the abstracted groundwater against available stocks and flows. For instance, some countries restrict access to groundwater data as they are considered of strategic importance (Jha and Chowdary 2007; Kumar 2002). Open access to groundwater data for research purposes should be facilitated globally. Furthermore, it is essential to obtain more data on how the abstracted groundwater is used. For instance, Wada et al. (2010) find that significant amounts of the abstracted groundwater globally are lost due to ineffective storage and treatment.

Holistic data on subsurface porosity are vital to more accurately determine the depth of groundwater occurrence (Arnell 2002). It is argued that the studies on porosity are best conducted at a smaller scale to improve accuracy. Likewise, literature review outlines the necessity to retrieve more data on the groundwater recharge and subsurface runoff (Margat and van der Gun 2013). Values on recharge and runoff are important for better understanding of how much groundwater can be abstracted without imposing a detrimental effect on its stocks. Research on this topic should start at smaller scales to ensure better data accuracy.

The volumes of usable groundwater internationally are restricted by quality which, in turn, depends on salinity, pollution, and, in some cases, hardness. Hence, new research agenda should supply reliable, detailed data on these topics, preferably extracted locally and then combined into detailed, global databases (Margat and van der Gun 2013). In addition, more studies should address the issue of interaction and exchange between surface- and groundwater as its magnitude can be significant but yet poorly understood (Palakodeti et al. 2009; Wada et al. 2010). The topics of groundwater quality and groundwater interaction with surface water bodies are interrelated as, for instance, sea water can penetrate groundwater stocks in coastal regions, thus negatively affecting their quality.

More research is also required in the areas that are traditionally seen as well explored, but prove to be insufficiently examined at closer analysis. For instance, the geography of global groundwater distribution calls for more comprehensive understanding (Margat and van der Gun 2013), especially in the context of groundwater stocks that occur under the ocean floor. The study by Post et al. (2013) indicates that these can be significant and should be included into the estimates of global groundwater resources. Likewise, Antarctica needs to be integrated into global groundwater assessments. While this continent has traditionally been excluded from analysis (see, for example, Shiklomanov 1997); recently, there has been evidence to suggest that Antarctica may host significant volumes of groundwater (Christoffersen et al. 2014), thus calling for more in-depth evaluation. While knowledge on the amount of groundwater stored under the ocean floor and in Antarctica is paramount for academic purposes, it is also significant from the practical standpoint as this groundwater can be considered a strategic global reserve and may exert so far unknown influences on climate change.

Furthermore, given the growing anthropogenic footprint on global water resources (Gleeson et al. 2012; Vörösmarty et al. 2004), it is vital to better understand the scope and the magnitude of man-made impacts on global groundwater. The new, previously unknown effects should be diligently researched. For instance, new evidence shows that hydraulic fracturing or fracking may have significant detrimental impacts on groundwater quantity and quality (Gordalla et al. 2013; Jackson et al. 2013; Stuart 2011) and these should therefore be examined in more detail given that the shale gas extraction is currently underway across the world (Hughes 2013).

Lastly, it is essential to better understand how the issue of climate change may affect the occurrence, distribution, and quality of global groundwater stocks and flows. While this area represents a growing research field, more studies are necessary. Detailed knowledge on this topic should help develop more effective adaptation and mitigation (Green et al. 2011; Pandey et al. 2011). Better understanding of the role played by global groundwater in the transition to a low-carbon economy is of particular importance (Younger 2014). For instance, the impact of biofuel production on global groundwater systems has been acknowledged but little is known about its magnitude and therefore more research is required (Uhlenbrook 2007).

#### Global Groundwater: Enhancement of the Methodological Base

Reliable methods play a crucial role in obtaining accurate data on global groundwater resources. It is argued that a new, ‘globalized’ research agenda should strive to promote application of reliable groundwater assessment techniques that have proven their potential in generating good-quality data at affordable costs. For example, more research is necessary on how the methods effectively utilized in soil mechanics could be adapted for making estimates of groundwater stocks and flows. The application of these methods should provide more accurate and reliable data on groundwater occurrence and dynamics. Furthermore, further isotope analysis can be used to supplement the techniques applied in soil mechanics. As shown by van Geldern et al. (2014), such techniques can help generate numbers on yet poorly quantified occurrences of groundwater, for instance under the ocean floor, thus contributing to more accurate assessments of groundwater stocks locally, regionally, and then globally.

The remote sensing approaches, such as GRACE (Rodell and Famiglietti 2002), have the potential to generate accurate estimates of variations in the volume of water storage compartments across the globe. The outcome can be used by modelling studies to obtain more reliable estimates on local and regional groundwater resources that, when combined, will derive a global picture. Similar to the case of data retrieval and access, it is argued that research on the application of different methods and techniques should first be conducted at smaller scales. Subsequent upscaling and assembly of smaller studies can enable larger outlooks. Collaboration between geophysics, geochemistry, and modelling can enhance the methodological base of the studies on groundwater resources and improve the overall accuracy of groundwater estimates.

#### Global Groundwater: Research to Enhance Policy-Making

The literature review showed that the outcome of groundwater studies is yet insufficiently embedded into policy-planning. For instance, Lavoie et al. (2013) emphasize the necessity to make better use of the data on groundwater resources for decision-making purposes. It is therefore argued that advances in ‘globalized’ research on groundwater stocks and flows should be more closely integrated into global, regional, and local water policies. There is evidence that detailed knowledge on groundwater resources can, for example, aid in developing viable instruments of economic regulation in the regions of freshwater scarcity (Esteban and Dinar 2013). Better integration of groundwater into decision-making will not only lead to the provision of more effective, research-informed policies but should also enhance the public recognition of the crucial role played by groundwater. Examples, such as the WHYMAP and IGRAC projects, should be more broadly utilized to achieve this goal. In this field, GIS methods are known as effective presentation, educational, and awareness raising tools due to the use of visual aids, such as maps and diagrams.

It is further argued that the issues of global groundwater stocks and flows should be better embedded into international policies on water management. These could aim at allocating specialized funds to developing countries that are often underlain by large aquifer systems with reduced groundwater monitoring networks. This would facilitate more research on groundwater storage and dynamics in the developing world, which in turn would provide reliable data to produce more accurate estimates of groundwater resources at different scales.

## Conclusions

Freshwater is a crucial environmental resource. Despite its global importance, stocks and flows are yet poorly quantified and large discrepancies exist in the estimates provided by the available literature. Within the various freshwater compartments, groundwater constitutes a vital part of usable global freshwater resources, but its volume assessments have the highest levels of uncertainty. The current knowledge on global groundwater reserves is often based on the outcome of research conducted in the last century and very few recent studies exist. New and more precise assessment techniques should find broader scientific application to generate more reliable estimates of global groundwater stocks and flows. Limited knowledge on global groundwater resources may lead to inexact policy-making and managerial decisions. In particular, they may cause the development of ineffective measures and strategies directed to mitigate the growing human-induced impacts on the depletion and pollution of groundwater, thus hampering its sustainable management.

Rising global demands for groundwater require more investment in its characterization and assessment, thus calling for a new stream in existing water research. This new research stream should have a more pronounced focus on groundwater and management of its stocks and flows at all geographical levels, thus reflecting the concept of ‘globalization.’ The new, more groundwater-focused agenda should aim at enhancing the accuracy of estimates on groundwater stocks and flows at local, regional and global scales. This can be achieved via the broader application of the most up-to-date assessment methods, such as remote sensing techniques, gravimetric and isotope analysis, and high-precision modelling.

The establishment and regular maintenance of regional and global data collection networks should be an integral element of the new research agenda. These networks should compile the outcome of local and regional studies on groundwater systems alongside the human interventions made to them. The results of studies on local and regional groundwater resources should find better integration into national and international water policy-making and management. This will aid in translating research findings from theory to practice and enhancing the public awareness on the international importance of groundwater. Ultimately, this should lead to more responsible consumption of this vital environmental resource.

## Footnotes

1 The estimates of water volumes and flows attributed to each of the water storage compartments differ considerably depending on the source of data. “Global (Ground) Water Estimates: A Critical review” section reviews this issue in detail. The figures cited here are the ‘averaged’ numbers reported in specialist literature.
